# COMOKIT: A Modeling Kit to Understand, Analyze, and Compare the Impacts of Mitigation Policies Against the COVID-19 Epidemic at the Scale of a City

**DOI:** 10.3389/fpubh.2020.563247

**Published:** 2020-09-24

**Authors:** Benoit Gaudou, Nghi Quang Huynh, Damien Philippon, Arthur Brugière, Kevin Chapuis, Patrick Taillandier, Pierre Larmande, Alexis Drogoul

**Affiliations:** ^1^UMI 209, UMMISCO, IRD, Sorbonne Université, Bondy, France; ^2^UMR 5505, IRIT, Université Toulouse 1 Capitole, Toulouse, France; ^3^ICTLab, University of Science and Technology of Hanoi (USTH), Vietnam Academy of Science and Technology, Hanoi, Vietnam; ^4^College of Information & Communication Technology (CICT), Can Tho University, Can Tho, Vietnam; ^5^WHO Collaborating Centre for Infectious Disease Epidemiology and Control, School of Public Health, Li Ka Shing Faculty of Medicine, The University of Hong Kong, Hong Kong, China; ^6^UMR 228, ESPACE-DEV, IRD, Montpellier, France; ^7^UR 875, MIAT, INRAE, Toulouse University, Toulouse, France; ^8^UMR 232, DIADE, IRD, University of Montpellier, Montpellier, France

**Keywords:** COVID-19, agent-based modeling (ABM), epidemiological modeling, GAMA platform, computer simulation (CS)

## Abstract

Since its emergence in China, the COVID-19 pandemic has spread rapidly around the world. Faced with this unknown disease, public health authorities were forced to experiment, in a short period of time, with various combinations of interventions at different scales. However, as the pandemic progresses, there is an urgent need for tools and methodologies to quickly analyze the effectiveness of responses against COVID-19 in different communities and contexts. In this perspective, computer modeling appears to be an invaluable lever as it allows for the *in silico* exploration of a range of intervention strategies prior to the potential field implementation phase. More specifically, we argue that, in order to take into account important dimensions of policy actions, such as the heterogeneity of the individual response or the spatial aspect of containment strategies, the branch of computer modeling known as *agent-based modeling* is of immense interest. We present in this paper an agent-based modeling framework called COVID-19 Modeling Kit (COMOKIT), designed to be generic, scalable and thus portable in a variety of social and geographical contexts. COMOKIT combines models of person-to-person and environmental transmission, a model of individual epidemiological status evolution, an agenda-based 1-h time step model of human mobility, and an intervention model. It is designed to be modular and flexible enough to allow modelers and users to represent different strategies and study their impacts in multiple social, epidemiological or economic scenarios. Several large-scale experiments are analyzed in this paper and allow us to show the potentialities of COMOKIT in terms of analysis and comparison of the impacts of public health policies in a realistic case study.

## 1. Introduction

### 1.1. Context: The COVID-19 Pandemic

In December 2019, human infections by an unknown agent causing pneumonia were reported in Wuhan, China (1). The infectious pathogen, later known as SARS-COV-2, is a novel coronavirus responsible for causing the new COVID-19 disease.

While the first human cases appeared to be related to a seafood market, the following cases were not, indicating that SARS-COV-2 is capable of sustained human-to-human transmission (2). This preliminary investigation of the Wuhan outbreak in mid-January reported a baseline reproductive index (R0) of 2.2, meaning that the introduction of an infected individual into a fully susceptible population would result in an average of 2.2 additional infections. This strongly suggests that outbreaks could have grown exponentially if interventions and containment strategies had not been put in place early enough.

Given the initial lack of knowledge about the COVID-19 disease, the differences in preparedness, practices and cultural background of their populations, countries have naturally chosen different intervention policies to fight the pandemic. For instance, South Korea decided to move to massive drive-through virus testing programs after a fast increase of the number of infected cases (3), while France chose a late lockdown of the whole country [see (4) for an interesting overview of the strategies of 11 EU countries]. China imposed a lockdown to the most impacted city, Wuhan (followed by a lockdown of the entire province of Hubei) and implemented a strategy of contact tracing through the use of a smartphone application giving the exact location of an individual through time, allowing fast identification of contacts of an infected case (5). In Hong Kong, the fast implementation of border restrictions, isolations and quarantine, coupled with school closures and social distancing, has been shown really effective to reduce the transmission (6). Singapore initially chose to keep schools open, but performed health checks, reduced social gatherings, canceled large scale events, and traced contacts of infected cases, allowing the public to know the exact location of a known case once reported (7). Finally, Vietnam quickly chose to limit exchanges with China and applied very localized policies: for every identified infected individual, authorities tracked all the persons in contact with it and quarantined them. They also decided very early to lockdown full communes (e.g., Son Loi and Ha Loi in the province of Vinh Phuc) (8–10), an intervention similar to China but at a much smaller scale.

### 1.2. Proposal: An Agent-Based, Spatially Explicit, Modeling Kit

The wide range of possible interventions makes it extremely difficult to decide which ones are most appropriate in a given context. In this regard, computer modeling is an invaluable tool for exploring a range of intervention strategies *in silico* before the potential field implementation phase (11–13). It has been widely used, for example, to justify public health policies based on locking down entire populations (6, 14). However, while classical compartmentalized epidemiological models (15) or highly simplified individual-based models (16) seem to be relevant at the scale of an entire country, they are paradoxically not relevant at smaller scales, where it is of utmost importance to be able to accurately predict the impact of localized interventions. As a matter of fact, when an intervention is applied on a small population, the individual and social heterogeneities in terms of social or economic characteristics, medical profiles (17), spatial distribution (18), behaviors, opinion, or compliance to the public rules (19), are crucial factors to take into account in models. Moreover, among these features, some might remain constant (e.g., spatial distribution) but others can evolve during the intervention itself (e.g., compliance), making it difficult to approximate them with average values: models that only consider the evolution of the epidemic through the interactions between aggregated variables (representing compartments or other subsets of the population) are unable to represent these heterogeneities, let alone their evolution, and thus to use them for analysing, comparing, or even proposing possible interventions.

The urgent need of tools and methodologies that enable fast analysis of the effectiveness of the responses against COVID-19 across different communities and contexts, including small-scale ones, made us adopt an approach based on the design and simulation of agent-based computational models (20), where the profiles of people and households, their interactions, their evolution in time and space, are explicitly represented and serve as a basis for describing the dynamics of the epidemic. This is a “complex systems” perspective (21), where this dynamics is not only the result of a transmission mechanism, but also that of the non-linear interactions between actors with complex relationships and mechanisms across numerous levels of organization, which act and interact with each other and with their environment.

This has led us to design COMOKIT (COVID-19 MOdeling KIT) based on the agent-based modeling and simulation platform GAMA (22). As stated in Drogoul et al. (23), COMOKIT follows a set of principles:

- be as close as possible to public decision making by having the possibility to answer to concrete questions;
- be based on a detailed and realistic representation of space (public health policies are also predominantly spatial);
- rely on spatial and social data that can be collected easily and quickly;
- be generic, flexible, and applicable to possibly any case study;
- be trustable by relying on inner mechanisms that can be isolated and validated separately;
- be open and modular enough to support interdisciplinary cooperation;
- offer an easy access to large-scale experimentation and statistical validation by facilitating the exploration of its parameters;

This article is organized as follows. In section 2, we propose a rapid state of the art, which allows us to point out the limitations of existing models (whether mathematical or agent-based) in terms of decision support and realism in representing the impacts of interventions against COVID-19. Section 3 then presents the main structure and processes of the COMOKIT model, designed not only to overcome these limitations but also to provide a basis from which more comprehensive models can be built. In section 4, we present a set of experiments carried out on COMOKIT with two ambitions: the first to show its dynamic characteristics (in terms of sensitivity to certain parameters, stochasticity and the need for replication), the second to show its potentialities in terms of studying and comparing the impact of public health policies in different scenarios. On the basis of these very encouraging initial results, section 5 concludes by listing some of the limitations of version V1.0 of the model and presenting its prospects for evolution and application to different contexts.

## 2. State of the Art

Several modeling studies have been undertaken at very early stages of the pandemic in order to study the impact of different policies against COVID-19 and to better prepare public health systems. Most of them relied on well-known mathematical models. As a matter of fact, at least in epidemiology, mathematical models are tools that can be developed very rapidly to answer a limited range of questions in critical and urgent situations. For example, such a model, using the meta-population of different cities represented by a Susceptible Exposed Infectious Recovered (SEIR) compartment model, was developed in less than a month to predict the spread of COVID-19 in a region or country and estimate the number of cases exported from Wuhan through human mobility and flights (24). This model was useful in showing whether and to what extent cases were likely to occur in areas other than Wuhan. Another mathematical model was used to represent the risk of virus introduction and the effectiveness of symptom screening in travelers (25). This probabilistic process model showed that, because of asymptomatic and pre-symptomatic infections, symptom screening alone was not sufficient to prevent the introduction of infected persons. Mathematical models have also been used to study control and non-pharmaceutical interventions in Europe, Wuhan and more abstract contexts (4, 26–28). For example, a model was designed taking into account the different contacts between the age groups represented in the SEIR compartments and examining the effect of control strategies implemented in Wuhan (27). Another model studied the effect of lockdown in European countries, assuming that the effect was the same regardless of the country of implementation, using a Bayesian approach (4). Health care capacity in the United States has also been studied using compartmentalized models representing individuals in the same age category in different states with different age-contact matrices (29). Finally, mathematical modeling was also applied prospectively to study the post-pandemic situation, examining seasonality and herd immunity (30, 31).

While mathematical models are particularly useful for rapid response and when there is a high degree of uncertainty in the different parameters, they also assume a certain homogeneity of individuals in a population, which can be a weakness when it comes to representing dynamics that rely heavily on individual aspects. While the use of age matrices in different compartmentalized models has countered this phenomenon, taking into account the fact that older populations appear to have a higher risk of developing a severe and more fatal form of the disease (4–6) while children are less likely to develop symptoms (32, 33), these models are still unable to take into account heterogeneities between individuals in terms of social relationships, behaviors, and attitudes toward the disease (34).

For example, intervention policies, such as lockdown are effective when everyone acts in accordance with policy statements. However, studies show that age groups may respond differently to containment, which may increase the risk of infection for that particular group (35, 36). This is particularly important because super-spreading events (infections of several people by one individual) have been reported in several locations (37). It is therefore essential to add complexity and heterogeneity in the models in terms of social relationships, spatialization, and individual characteristics. Although more complex to design and to explore (because of a generally more stochastic approach) than mathematical models, individual-based models have begun to be used to study COVID-19.

In Hellewell et al. (38), an individual branch process model is proposed to examine the possibility of preventing the introduction of the disease into a totally disease-free population by applying isolation and contact tracing. Interventions have also been studied in different contexts. For example, in Ferguson et al. (6), an ABM representing the population with different contact settings (school, work, home, etc.) for high-income countries has been designed to study the impact of different interventions to mitigate epidemics, including social distance, isolation of cases, quarantine and school closure. The model took into account spatialization but also individual characteristics to represent the risk profile, using the number of patients in intensive care units (ICUs), hospitalizations and deaths as indicators. However, the possibility of environmental transmission was not taken into account in the model. Indeed, several studies have shown that the virus can survive in the environment and on different types of surfaces (39, 40), possibly leading to environmental contamination and transmission, but also to nosocomial infections (41, 42). This type of transmission has already been reported in other coronaviruses, such as SARS and MERS (43, 44), and infections of several health care workers have also been reported (45). In addition, evidence of the viability of aerosolized virus transmission has also been provided (46). Another limitation of this model is that it does not account for hospitalizations, although it is known that some deaths are due to lack of hospital capacity. Finally, no information on recreational activities was represented, although bars, restaurants, nightclubs, cinemas and the like can be important contamination sites (47).

In Wang et al. (48), another model is presented, representing 2,000 people in four different states (susceptible-latent-infectious-removed) and examining a possible set of interventions, such as personal protection, isolation and quarantine, containment and social distance, and their cost-effectiveness after importation of infected cases. Again, intensive care and hospitalizations were used as indicators, but sociological aspects were not represented in this model. Transmission occurred in the community without taking into account households, workplaces or other social gathering events known to facilitate the spread of the disease, and again, no environmental contamination was represented.

Modeling of pandemic transmission and control was also the objective of another study using ABM in Australia (19). In this model, interventions, such as school closures, travel bans, social distancing and case isolation were studied using an influenza-derived model representing a synthetic population of 24 million individuals with their own characteristics and social context. Nevertheless, no environmental contamination was represented due to the large scale of the model, which prevents the representation of buildings and other places. In addition, the model did not take into account the possibility of leisure activities, which can be explained by the fact that each stage corresponds to several hours (day and night periods). Finally, no dynamics concerning hospitalization capacity were represented.

Finally, another agent-based model, derived from influenza, was used in a study in Singapore (11), representing transmission during 12-h cycles for a set of buildings visited by infected persons. Again, school closure, quarantine and isolation of cases were studied, with an interesting aspect of the model being the focus on high-risk locations. However, as with the two models previously mentioned, no recreational activities were represented, as the temporal representation was done by day and night steps. In addition, environmental contamination was also not taken into account.

This rapid state of the art, far from being exhaustive due to the proliferation of more or less similar models, nevertheless makes it possible to highlight several limitations of existing models in terms of decision support:

- The limited and usually not flexible representation of individual activities does not allow these models to faithfully reproduce many social dynamics known to be at risk in terms of transmission: group leisure activities (karaoke, dance halls, restaurants, bars, etc.), groups at school or in companies, religious celebrations, etc.
- The often too large time step (day or half-day) cannot account for the shorter contacts or interactions that nevertheless constitute the bulk of our daily interactions. The resulting “averaging” effect erases any representation of the behavioral heterogeneity of individuals.
- In these models, individuals, even if their behaviors are different, are assumed not only to react in the same way to health authorities' injunctions, but also to do so in the same way regardless of when these injunctions are issued. However, a crucial point in the implementation of intervention policies is precisely to know how to anticipate the population's acceptance or rejection, and to be able to measure the effects of habituation, exasperation, or even revolt toward these policies.
- No environmental transmission is envisaged in any of these models, which raises the problem of their realism, especially when they take as a case study urban environments, where the opportunities for transmission through synthetic surfaces handled by many people (lifts, public transport, vending machines, handrails, counters, etc.) are legion.

COMOKIT has been primarily designed to meet these limitations. The following section presents version V1.0 of the model^1^ in more detail.

## 3. Model

### 3.1. Overview of the Model

COMOKIT aims to simulate and compare the application of policies to mitigate the spread of COVID-19 at the scale of an urban area, with the disease being modeled at the individual scale. Its objective is to answer questions, such as: Is the containment of a neighborhood more effective than that of an entire village? Does school closure reduce transmission peaks? How does the wearing of masks affect the dynamics of the epidemic? What should be the ideal duration of containment? What proportion of the population should be allowed to engage in activities during a containment?

COMOKIT combines a sub-model of direct person-to-person transmission, a sub-model of environmental transmission through the built environment, a policy design model, and an agenda-based model of mobility and occupation of people at a rate of 1 h. A key point is that it allows the representation of heterogeneities in individual characteristics (gender, age, household), agendas (based on social structures, available services or age categories), social relations behaviors (e.g., compliance with policies), and response to COVID-19.

### 3.2. Description of the Model Entities

**Figure 1:**
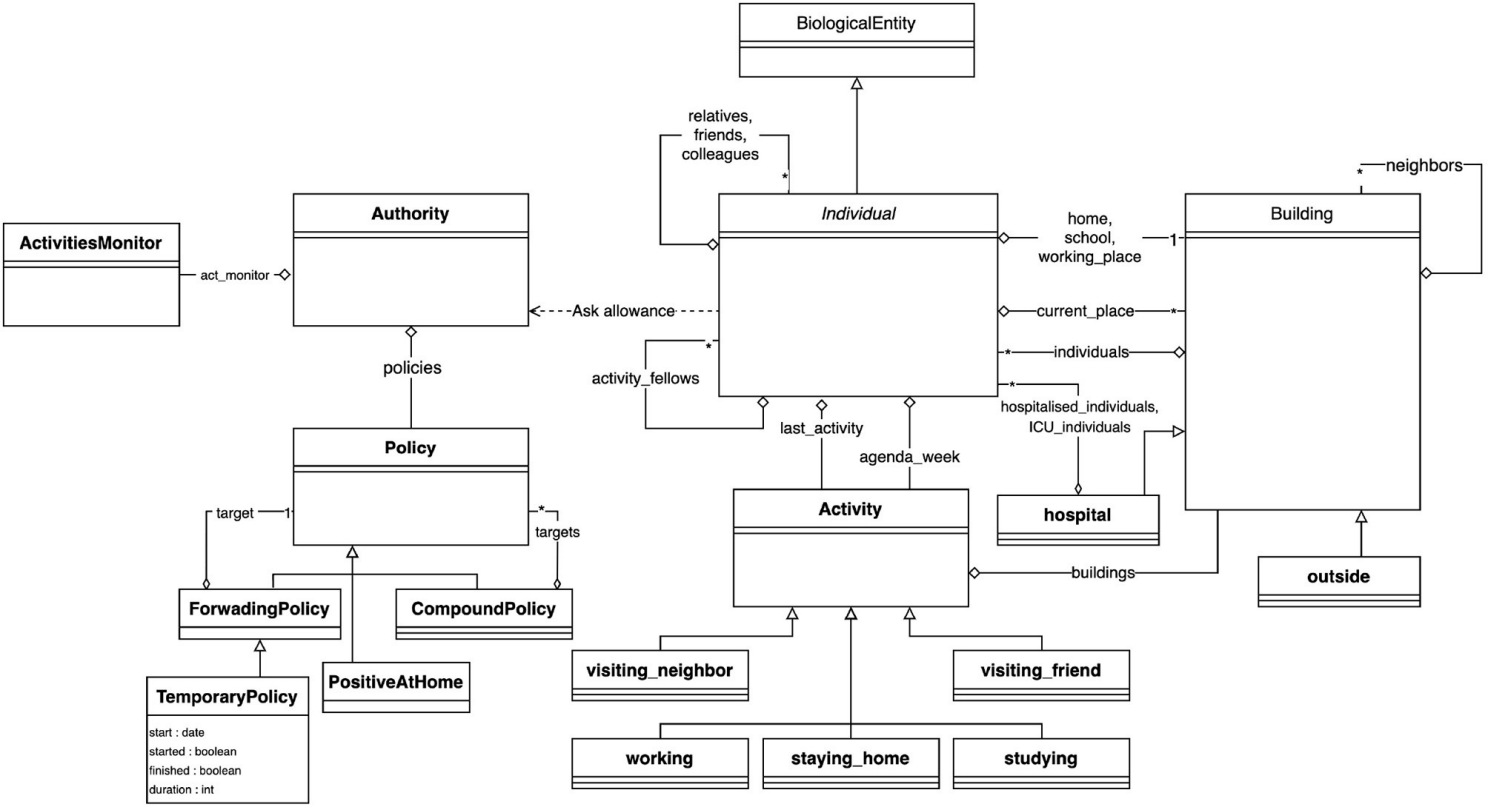
Class diagram of the COMOKIT entities.

The central entity of the model is the *Individual* type (or species) of agents: it represents the individual inhabitants of the area under consideration with their individual characteristics (age, sex, occupational status) and their epidemiological status, whether they have been tested, and other individual epidemiological values (e.g., latent_time, infectious_time …more details in section 3.3.3). They carry out their daily activities (e.g., going to work, school, shopping, eating out, etc.) according to their personal agenda. This agenda is a set of generated activities that can be shared by several people (for example, going out to eat with friends), depending on the age and family status of the *Individual* agent. Attributes of *Individual* agents include their parents (their family, which in our model corresponds to the other *Individual* agents living in the same apartment in a *Building*), friends (with whom they can share activities), colleagues (co-workers or classmates), and their home, workplace, and school *Buildings*. An overview of the structure of the model is presented in the form of a UML class diagram in Figure 1.

*Building* agents are spatial entities where Individual agents can perform an Activity, which depends on the type of *Building*. Two special types of Buildings have been defined because they play an important role in the simulation: The Outside, which houses the activities performed by individuals outside the modeled area, and the Hospital, where sick Individual agents with critical symptoms can be contained and treated. In order to take into account the possible transmission of the virus through the environment, all *Buildings* are equipped with a viral load, which can be used by the epidemiological sub-model (see section 3.3.3).

Individuals' hourly behaviors are determined by their agendas, which associate Activities with hours. Individuals have preferences for certain types of Activities that can be set according to their age and gender: for a leisure activity, a child may prefer to go to a play center while an older person may prefer to go to the cinema. The Building where Individuals carry out an Activity can be chosen at random (uniformly), as the closest, or according to a probability (negative function of distance and positive function of the area of the target place). COMOKIT also defines a number of specific Activities to represent some classical ones: visiting_a_neighbour, working, staying_at_home, studying, visiting_a_friend. Of course, custom activities can also be created from the generic Activity species.

In COMOKIT, particular attention is paid to policies that change the behavior of the population in order to reduce contact and thus infections between people: an Individual's ability to engage in a particular Activity is limited by the authorization of the Authority agent. Authorization to engage in specific activities depends on the Policy adopted and managed by this Authority. Examples of Policy include total containment, schools closure, working places closure …These Policies may be limited to a given area (using SpatialPolicy) or may be more or less tolerant (for example, containment may be complete or complete but for some people, or a certain percentage of the population, using PartialPolicy).

### 3.3. Description of the Model Processes

#### 3.3.1. Initialization

A simulation is initialized by creating *Building* agents from shapefiles, *Authority* and *Policy* agents, and setting other parameters from data files. The *Individual* agents with their demographic attributes are created from a synthetic population generator [either an *ad hoc* generator coded in the model or by the Gen* generator using available data (50)]. Agendas are created using an *ad hoc* generator: they are composed by seven daily agendas depending on the *Individuals*' age and employment status: students and workers have an agenda composed of working days and leisure days (i.e., a day with activities different from working, learning or staying home); retired and unemployed *Individuals* have an agenda full of leisure days. *Individuals* that are too young have an empty agenda. The choice of activities outside of work and study will depend on the age and gender of the *Individual*. It is indeed possible to parameterize (through a CSV file) the fact that young people will, for example, favor leisure activities while elders will favor shopping activities. For each activity, a list of fellow *Individual* agents sharing the same activity can be defined to represent for example a group of friends or colleagues eating at the same table in a restaurant. Lastly, the simulation is initialized with N (a parameter) infected (but not yet infectious) *Individual* agents.

#### 3.3.2. Process Overview and Scheduling

The dynamics of the model is entirely represented by three interconnected but nevertheless independent sub-models: **ESM**, the epidemiological submodel (which combines infection, hospitalization and transmission processes), **ASM**, the activity submodel, and **PSM**, the policy submodel (which combines application and adoption processes). The simulation step is set to 1 h.

A simulation step starts by the evolution of the viral load in a building (it decreases over time, before disappearing). Then the Individual agents first evaluate whether they are infected. If they are, they may infect other Individuals and/or contaminate the current building in which they are located. Depending on their updated epidemic status, individuals will revise their behavior (e.g., wearing a mask) and execute their daily activities: they find the activity corresponding to the current hour, ask the Authority whether they are allowed to execute it and act in accordance. Finally, the Authority agent checks its current Policy and tries to apply it (e.g., executing a mass testing campaign).

#### 3.3.3. ESM, the Epidemiological Submodel

As the virus is capable of surviving for long periods in the environment (39, 46), we consider two possible pathways of viral transmissions: either human-to-human transmission, through interactions between neighboring *Individual* agents, or, because of the potential persistence of the virus in the environment, through contacts between co-located *Individual* and *Building* agents, the latter of which being provided with a dynamic viral load (increased by the long-term co-location of infectious individuals, and decreasing according to some decay).

**Figure 2:**
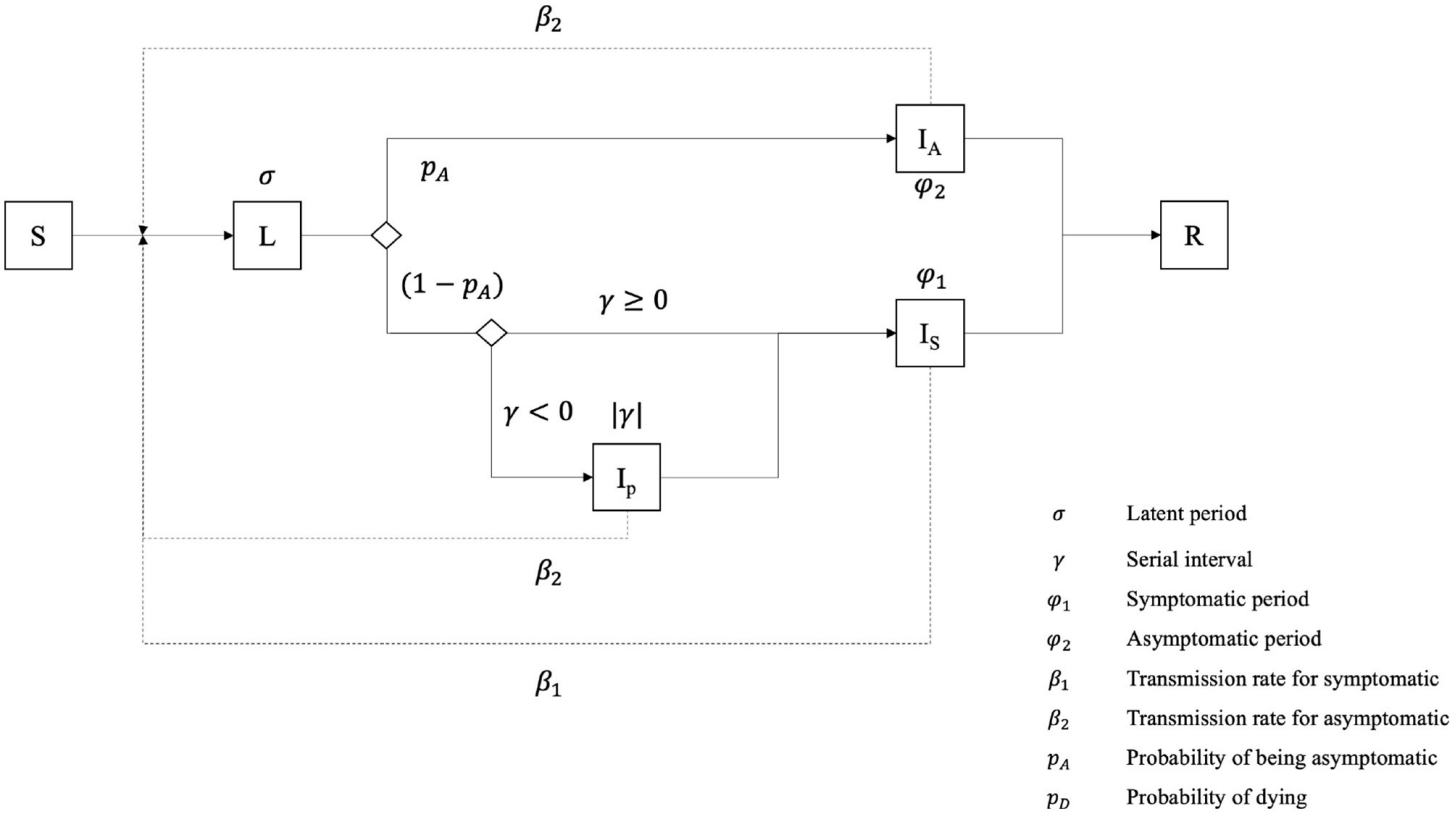
Epidemiological model of an Individual agent.

In our model, the disease-related state of the Individual agents follows a slightly modified SEIR model (15) (Figure 2). First, we assume that the whole population starts the simulation in the **Susceptible** state (**S**): as this is an emergent disease, nobody is immunized. When an *Individual* is in contact with an infectious *Individual* or located in an infected *Building*, it can become infected and move to the **Latent** state (**L**) (a renaming of the traditional *Exposed* compartment), depending on the success of the transmission, defined by the probability for one *Individual* at a given step to be infected by an infectious *Individual* in the same *Building*, or by a *Building* with a positive viral load.

Once the latent period is expired, an *Individual* transitions to one out of three possible infectious states (whereas the traditional SEIR model contains only a single one): it can become **asymptomatic** (IA), **pre-symptomatic** (IP) or **symptomatic** (IS). If the *serial interval* value is negative, it becomes **pre-symptomatic** for a short time, equal to the absolute value of the *serial interval*, before transitioning to the **symptomatic state**. The *Individual* remains in these states during the *serial interval* (for **pre-symptomatic** ones) or the *infectious period* for **symptomatic** and **asymptomatic** ones. Finally, we consider that **asymptomatic** and **pre-symptomatic** Individuals share the same *transmission rate*, i.e., the chance of infecting a neighboring susceptible *Individual*, while **symptomatic** agents have a much higher one.

After the *infectious period, Individual* agents become **Removed** (**R**): they are not infectious anymore and fall into one out of two sub-compartments, **Recovered** (**RR**) or **Dead** (**RD**).

**Figure 3:**
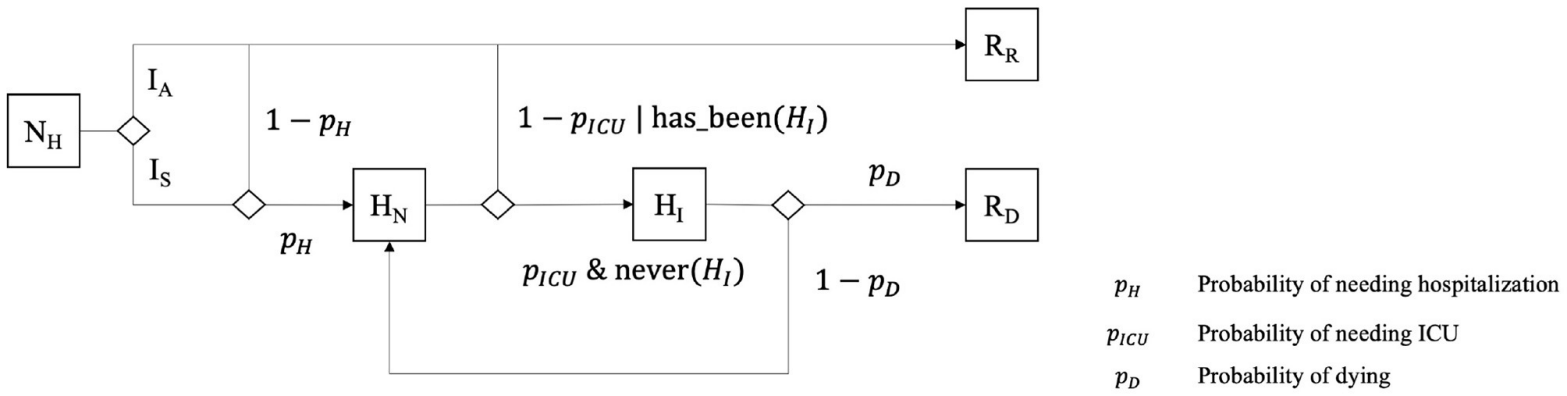
Additional states of Individuals, used and manipulated by the *HospitalizationPolicy* when it exists.

During their infectious period, symptomatic individuals can go through different clinical states: not needing hospitalization (**NH**), needing hospitalization (**HN**) and needing ICU (**HI**). Previous **asymptomatic** agents (la) become directly **Recovered**, as we assume that they cannot die from COVID-19 without showing symptoms, whereas symptomatic ones have a probability to recover or die (Figure 3). This probability depends on the (given) severity of the disease for the age category of the agent and the care it has been provided with (i.e., hospitalization and ICU). We consider that *Individuals* needing intensive care will become **Dead** if they do not get it. On the contrary, symptomatic *Individuals* that do not need intensive care (i.e., not needing hospitalization or needing hospitalization without intensive care treatment) become **Recovered**.

It is important to note that **ESM**, despite the fact that it is a more detailed model than most of those used in agent-based models (19, 48), makes certain assumptions, some of which are shared with other epidemiological models because of a lack of knowledge about the disease, others because we assume that they have no influence on the model itself.

1. Effective contact rate
  (a) Presymptomatic and asymptomatic individuals share the same contact rate
  (b) The contact rate does not differ during the infectious period.
  (c) Masks do not deliver any protection, but rather reduce the effective contact rate of an infectious individual and its viral release in the environment.
2. Environmental transmission
  (a) *Individuals* can be infected by a contaminated environment, and for a maximal viral contamination in one building, the effective contact rate is the same as one infectious *Individual*.
  (b) The viral release of an *Individual* in its environment (in our model, in *Buildings*) is the same for all infectious *Individuals*
3. Homogeneity of the population
  (a) The sex of individuals does not have any impact on the epidemiological model.
  (b) The age of individuals does not have any impact on the incubation period, the proportion of asymptomatic cases, and the effective contact rate for human to human and environmental transmission
  (c) Asymptomatic and symptomatic individuals share the same infectious period distribution
4. Recovery and death
  (a) Recovered Individuals are totally immunized against the infection.
  (b) Infection can lead to death only for *Individuals* expressing a need for intensive care.
  (c) Testing is performed only for virus isolation, not antibodies, therefore recovered people are not considered positive.

#### 3.3.4. ASM, the Activities Submodel

The *Individual* agents in COMOKIT are an extremely simplified representation of their actual counterparts; their daily activities are ultimately only the dynamic support of their role as disease spreaders. These activities, as discussed in section 3.3.3, are organized in the form of a weekly agenda that can distinguish between days off and days worked, and provides an hour-by-hour activity for all the agents. Once the weekly and daily agendas are created, at each simulation step, and unless they have already been enrolled in a collective *Activity, Individual* agents obtain the *Activity* corresponding to the current day and time, request the authorization from the *Authority* agent to perform it, and find a nearby building associated with this specific *Activity*. *Individual* agents can also enroll certain agents to participate in the *Activity* (e.g., colleagues, friends …) who are expected to have a closer relationship, and therefore have a higher probability of being infected. Since we have set the time step at 1 h, we decided not to represent the movement itself from one place to another : *Individuals* are translated directly from their current location to the building chosen to perform their new *Activity*.

This last choice may appear to be a limitation (or at least a somewhat too restrictive assumption), but it is consistent with the scale at which we model the disease and the control policy. Public transport, which represents one of the main risks of transmission in large conurbations or on national and international scales, is not used in small or medium-sized towns, which are the current target of COMOKIT.

#### 3.3.5. PSM, the Policy Submodel

The *Authority* is responsible for implementing one or more mitigation policies that may impact the simulation in two ways: at each step, on one hand, the *Authority* may proactively perform certain actions, for example by conducting a given number of tests on the population, and on the other hand, each *Individual* agent asks the *Authority* whether it is authorized to perform a given activity.

We have chosen a modular approach to defining policies: a general policy is based on a small set of specialized, concrete policies (e.g., the *DetectionPolicy* that authorizes all activities, but performs tests at each step, or the *ActivitiesListingPolicy* that limits activities within a given set of authorized activities) that are composed using the composite (implemented by *CompoundPolicy*) and nesting (by *ForwardingPolicy*) design patterns:

- *CompoundPolicy* is a policy composed of a list of other policies. It applies the policies listed in order and allows an activity for a given individual if and only if it is allowed by all policies.
- *ForwardingPolicy* is a policy that embeds another policy and can change its enablement dynamically (for example, the specialized *SpatialPolicy* restricts the application of its target policy in a given geographical space, while *TemporaryPolicy* does it within a limited period of time).

Among the different policies delivered with the standard version of COMOKIT is the one that explicitly links to the epidemiological sub-model **ESM** (without being necessary for its operation). This is the *HospitalizationPolicy*, which depends on the existence of at least one hospital *Building* in the dataset, and which takes care of the *Individuals* that need to be hospitalized after a certain period of time following symptom onset, given by a distribution, and remain hospitalized until they are recovered or dead. Hospitalized *Individuals* are considered **Recovered** after having tested negative for a given number of consecutive days, and not showing symptoms (i.e., not being in the **Symptomatic** state).

The availability of these policies and the ease with which they can be combined make it possible to represent complex and realistic public policies. For instance, a “realistic lockdown” experiment was created to test the impact of a 60-days lockdown policy, in which positive individuals are not allowed to travel, others are only allowed to stay at home or shop, and only 10% of the total population is allowed to work. The policy of the *Authority* in this experiment is therefore constructed as a *TemporaryPolicy*, limiting the application of a *CompoundPolicy* within a 60-days period. This nested composite policy was composed of:

- A policy applying a given number of tests at each step of the simulation (this policy allows any activity).
- A policy prohibiting any activity other than shopping and staying home, nested in another policy that limits its application to 90% of the population (the remaining 10% are free to engage in any activity).
- A policy prohibiting any activity for those who have tested positive.
- The hospitalization policy described above.

### 3.4. Input Data

All input data files used to initialize a COMOKIT simulation are summarized in Table 1. In addition to the geographic data (buildings.shp, boundary.shp, and satellite.png), the files describe either the synthetic population of Individuals generated by an external tool or the parameters of the generators integrated in COMOKIT.

**Table 1:** Overview of the dataset.

**Data file**	**Data type**	**Description**	**Source**
Buildings.shp	GIS shapefile	Geometries of buildings, with their type and number of flats as attributes	OpenStreetMap, Google Maps, and hand digitalization from Google satellite image. For Ben Tre, the initial data come from the Land Use map (produced by the DONRE^*^ in 2010)
Population.csv	CSV tabular file	The synthetic population generated from a sample using the Gen* library. Each line corresponds to a single individual with age, sex, and household id	https://international.ipums.org/international/ https://www.gso.gov.vn/default_en.aspx?tabid=774
Population parameter.csv	CSV tabular file	The set of parameters to define the population of Individuals	See O.D.D. description for more details
Activity parameter.csv	CSV tabular file	The set of parameters to define the activity of Individual	See O.D.D. description for more details
Activity type weights.csv	CSV tabular file	According to the age (interval) and sex, the weight of the different activities	See O.D.D. description for more details
Building type weights.csv	CSV tabular file	According to the age (interval) and sex, the weight of the building type	See O.D.D. description for more details
Epidemiological Parameters.csv	CSV tabular file	The set of epidemic parameters for the COVID-19	Various sources from the literature (see O.D.D. description for more details)

* *DONRE stands for Department Of Natural Resources and Environment. This is a department of the Vietnamese Ministry Of Natural Resources and Environment*.

#### 3.4.1. Spatial Data

The initialization of the spatial environment of the model requires one main input: a shapefile describing the buildings of the studied area. This shapefile must obligatorily contain two attributes: the type of building, which will be used in the definition of activities (each type of activity will be linked to one or more types of buildings), and the number of apartments per building, which is used to locate the households inside (one household per apartment). COMOKIT provides a spatial data generation tool, allowing, from a spatial boundary given as a shapefile, to download existing OSM^2^ data of the area and put it in the right format so that it can be directly used in the simulations. The existing tool also allows the vectorization of images (e.g., GoogleMap) to enrich the OSM data.

#### 3.4.2. Demographic Data

The simulation initialization can use a CSV file describing the synthetic population, where each line (also called *record*) corresponds to a unique individual with age, gender, household identifier and employment status. The Gen* library (50) can be used to generate such a population file from an IPUMS^3^ open-access population sample file and the marginal distributions of the demographic attributes available on the given case study. The generation of this synthetic population follows the combinatorial optimization approach described in Williamson et al. (51). Among the various algorithms available, we chose a simple random draw in order to fit the actual population sample to a known aggregate distribution of attributes. This algorithm begins with a random population (containing the desired number of individuals) composed of households uniformly selected from the sample; we then exchange *n* records of the synthetic population with records drawn from the sample. This operation is repeated until either a minimum matching is obtained or a maximum number of iterations has been performed. In the different experiments we made, we found that the algorithms performed well with *n* equal to 5% of the population size and a maximum number of iterations equal to 100.

The obtained population contains only demographic variables. These are supplemented by built-in COMOKIT generators for location, social network, and agenda.

#### 3.4.3. Epidemiological Data

The epidemiological parameter file is a table of parameters. For each parameter, the following values are provided: (i) the name of the parameter, (ii) the lower limit of the age category, (iii) whether the value of the parameter is given or whether it is to be chosen from a given probability distribution (and in this case the distribution considered), (iv) its value (if of a given value type) or the first parameter of the distribution, and (v) the second parameter (of the distribution). These data contain in particular the parameters that will make it possible to specify, in different case studies, the human-to-human transmission (within households, during activities) and environmental transmission processes.

### 3.5. Outputs

**Figure 4:**
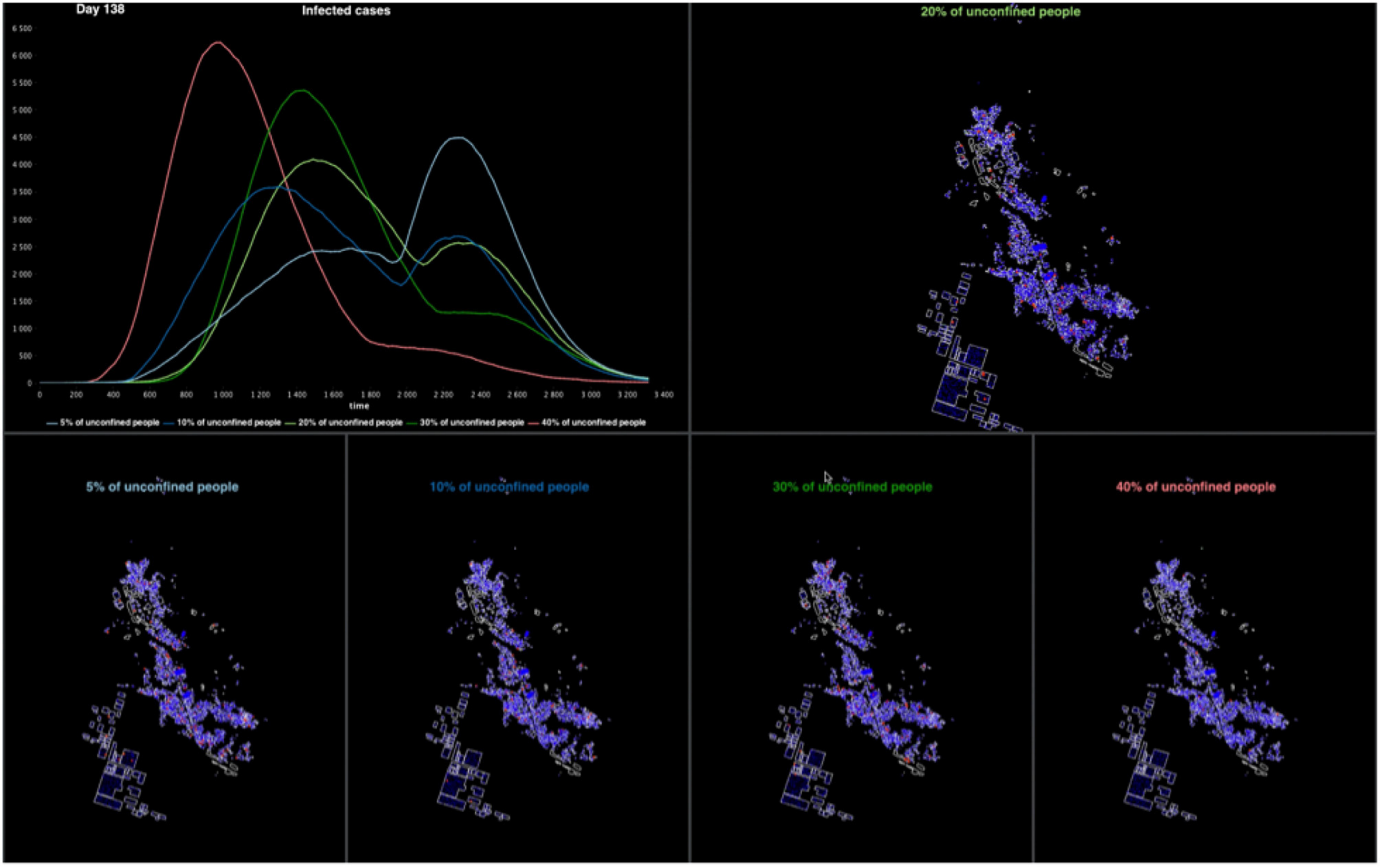
Example of the graphical interface of a COMOKIT experiment on Son Loi commune. The experiment compares five simulations with different numbers of unconfined people.

Much of the rapid assessment of a model's relevance depends on its ability to display results in a way understandable by its designers, programmers and users. When COMOKIT is used in the form of a dashboard or a demonstrator, the user interface of the simulations that each experiment runs can be completely defined and specialized according to the needs of its users. As the model was primarily designed to evaluate and compare policies, most experiments run several simulations in parallel with different parameter values. The user interface then contains a display of the spatial evolution of the disease for each parameter value and a graph plotting the evolution of the number of infected individuals over time. Figure 4 shows an example of such an interface considering five different proportions of unconfined individuals. It is also possible to display decreases in activity for different types of activities (compared to a baseline where no policy would be applied). Many other visualizations are possible, both in 2D and 3D, using the declarative approach proposed by GAMA. Some of them are provided in the model as a base, but can be enriched according to the needs of the users in order to compose real dashboards.

## 4. Experiments

In order to illustrate how COMOKIT can be used, we conducted a series of experiments for Son Loi Commune in Vinh Phuc Province, Vietnam. Son Loi is a rural commune of about 10,600 inhabitants and has 3,000 buildings of different types (houses, schools, temples, administrative buildings, industries.). Returning from a business trip to China, the first two cases were identified on January 17, 2020 (9). After the identification of nine other cases (on the 13th of February), the provincial authorities decided to lockdown the entire commune: the inhabitants were advised to stay at home and could not leave the commune; their state of health was checked daily and the authorities organized the supply of food and masks. After 18 days with no new cases identified, the lockdown was lifted on the 2nd of March.

To initialize the simulations, we first obtained spatial data on the buildings of the commune from the buildings present in Google Map and Bing data. The population input data file was generated using the Gen* library to produce a set of individuals grouped into households. We then used an approach based on combinatorial optimization to find a trade-off between maintaining the consistency of the microdata sample at the household and individual levels, while trying to match the census marginals (e.g., number of men/women, frequency of age category). In our case study, we used the IPUMS sample of individuals in households available for the whole Vietnam in 2014 (15% of the total population) with age, sex, household identification and employment status. Then, we randomly selected households with corresponding individuals in the sample to match the age and sex distribution at the individual level that we found in the 2019 Vietnamese census for Son Loi. The indicator chosen to assess the quality of the population is a normalized Total Absolute Error (TAE)^4^. The normalized TAE of the best synthetic population (that is used in the experiments below) is 0.1. This means that in the best generated population, when considering the distributions in each age and sex category, the number of individuals in the synthetic population differs on average by 10% from the aggregate census count.

As far as epidemiological parameters are concerned, most of them come from the literature. A preliminary calibration step is nevertheless necessary to make the disease transmission rate matching with data available on the considered case study: the “Successful_contact_rate_human” (the main parameter impacting the transmission between human beings) is computed given the R0 of the epidemic in the considered area and the average number of contacts between people in the simulation^5^.

We used the default value for all the other parameters (see the O.D.D. description of the COMOKIT model for the complete set of values, c.f. section 3).

### 4.1. Stochasticity Sensitivity Analysis

In a first experiment, we analyze the impact of the randomness of the simulations on the results and in particular on the dynamics of the epidemiological status of *Individuals*. The main objective is to find a threshold value of replications beyond which an increase in the number of replications would not imply a significant marginal decrease of the difference between the results. To do this, we compare the global incidence (defined here as the number of new infected individuals per time step), and the number of *Individuals* recovered and dead, between replications of the simulation. Incidence dynamics are not expected to be smooth since the number of new infections depends on the contacts between *Individual* agents, and those do not have much contact when staying at home during the night. We undertake this exploration with the simplest possible scenario, i.e., a free spread of the disease without containment and two infected individuals at the beginning of the simulation. We perform 500 replications of such a simulation and compare the variability of the results for the first 25, 50, 100, 250, and 500 replications.

**Figure 5:**
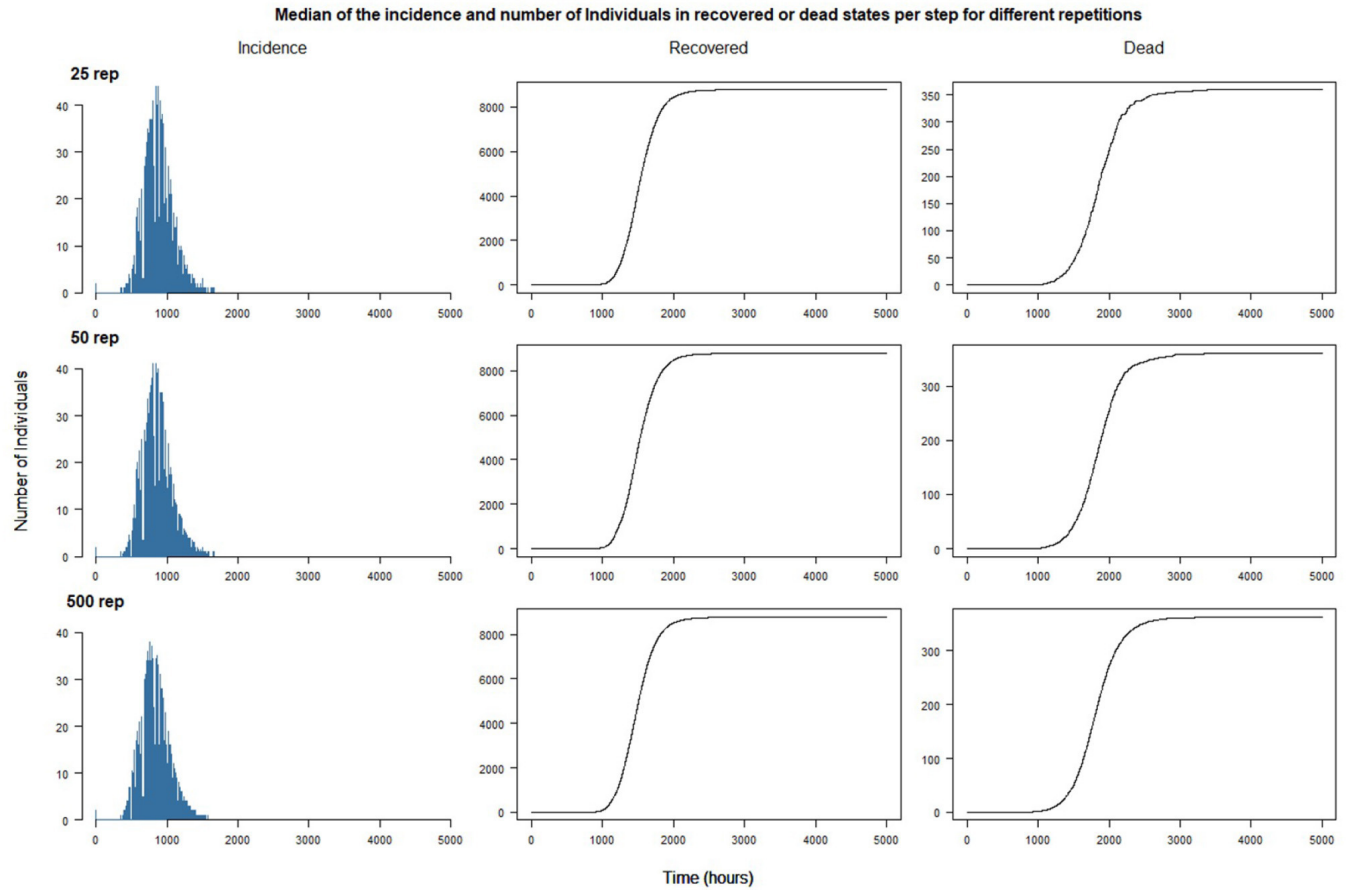
Median of the incidence and number of individuals in recovered or dead states per step for 25, 50, and 500 different repetitions.

In Figure 5, we plot the median value (over the replications), by time steps, of the incidence (first column), the number of individuals recovered (middle column) and dead (last column). The shapes are as expected for a SEIR-like epidemiological model of the disease: the incidence increases exponentially until a peak before decreasing to 0, and the number of Recovered and Dead Individuals increase until a time step where they become constant.

The results suggest that increasing the number of replications beyond 25 does not have a great impact on the aggregate trend of the simulated epidemic: the curves soften as the number of replications increases, but the patterns remain the same. One of the reasons is certainly the absence of interventions outside the introduction of the first two cases: the dynamics of propagation is ultimately only marginally influenced by the usual activities of the agents. On the other hand, we can expect the simulations to show quantitatively and qualitatively different results, or greater variability, when interventions will be introduced (see next section).

**Figure 6:**
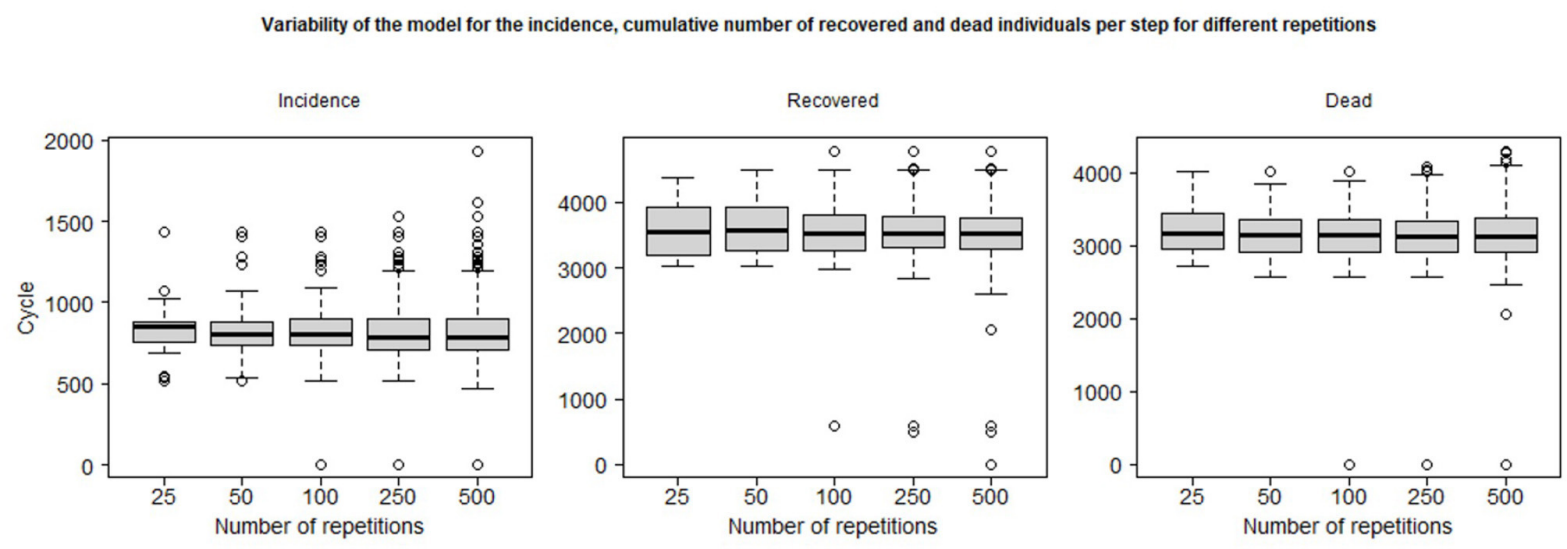
Whiskers plots and minimum/maximum excluding 1.5 IQR outliers of the simulation step of the maximum of the incidence, and the minimum step to reach the maximum of the cumulative number of recovered and dead individuals per step for different number of repetitions.

Figure 6 plots the simulation steps for the maximum of incidence and the steps to reach the maximum of the number of Dead and Recovered Individuals: it shows the median (black line), the second and third quartiles (the box) and the minimum and maximum peak cycle (whiskers) excluding *outliers* (simulation results that differ from the median by more than 1.5 times the IQR). We can observe that most of the simulations show a near peak cycle, between 500 and 1,500 for incidence, 3,000 and 4,500 for recovered individuals and 2,500–4,000 for deaths: this shows that the number of replications does not have a large impact on the aggregate outcome. However, after more than 100 replications, we have observed some simulations that show a very contrasted behavior: for example, when performing 500 replications, three simulations have their maximum number of agents recovered at less than 1,000 cycles, which means that the epidemic is not occurring or at least that the spread of the epidemic has been rapid and less impacting. The probability of “extreme” outcomes occurring (e.g., a long duration or complete absence of epidemic spread) is obviously positively correlated with the number of replications.

For the policy impact study presented in the following section, we decided to set the number of replicates at 50 in order to minimize the computation time required while trying to maintain realistic statistical properties, in particular the occurrence of extreme outcomes.

### 4.2. Comparison of Policies

In this section, we illustrate the possibilities of COMOKIT, on the same case study (Son Loi, Vietnam), for comparing the impacts of policies and analyzing their performance against a reference scenario where the virus would have spread freely. The simulations presented here are limited in number, as the objective is not to provide exhaustive results, for which an overall sensitivity study would have been necessary, and which would in any case make no sense for this particular case study, but to show what can be achieved with the simulator.

#### 4.2.1. Impact of Wearing Masks

The objective of this experiment is to evaluate the impact of wearing masks on the spread of the epidemic. While masks are still not recommended for the general population by the WHO and there is scientific debate on their use (52), a study has shown the ability of surgical masks to prevent the exhalation of respiratory viruses (53). In addition, asymptomatic and presymptomatic COVID-19 infections have been reported in different locations (54–57), and are suspected to play an important role in the persistence of epidemics (58). Therefore, the use of masks by the population could reduce the impact of presymptomatic and asymptomatic carriers by preventing them from releasing aerosols when they are not yet symptomatic, or droplets when they sneeze (not necessarily related to the disease). Due to past events related to respiratory diseases, such as SARS and influenza, people in Asian countries have been extremely cautious, wearing masks from the onset of the COVID-19 epidemic as a hygienic practice, even when people did not show any symptoms (52).

We therefore sought to assess the impact of the proportion of people wearing masks on the total incidence, the number of people recovered and the number of deaths. A comprehensive experiment exploring one parameter of the simulation (the proportion of individuals wearing a mask, taking a value between 0 and 1 and a step size of 0.25), was then launched. For each value of this parameter, we ran 50 replications.

**Figure 7:**
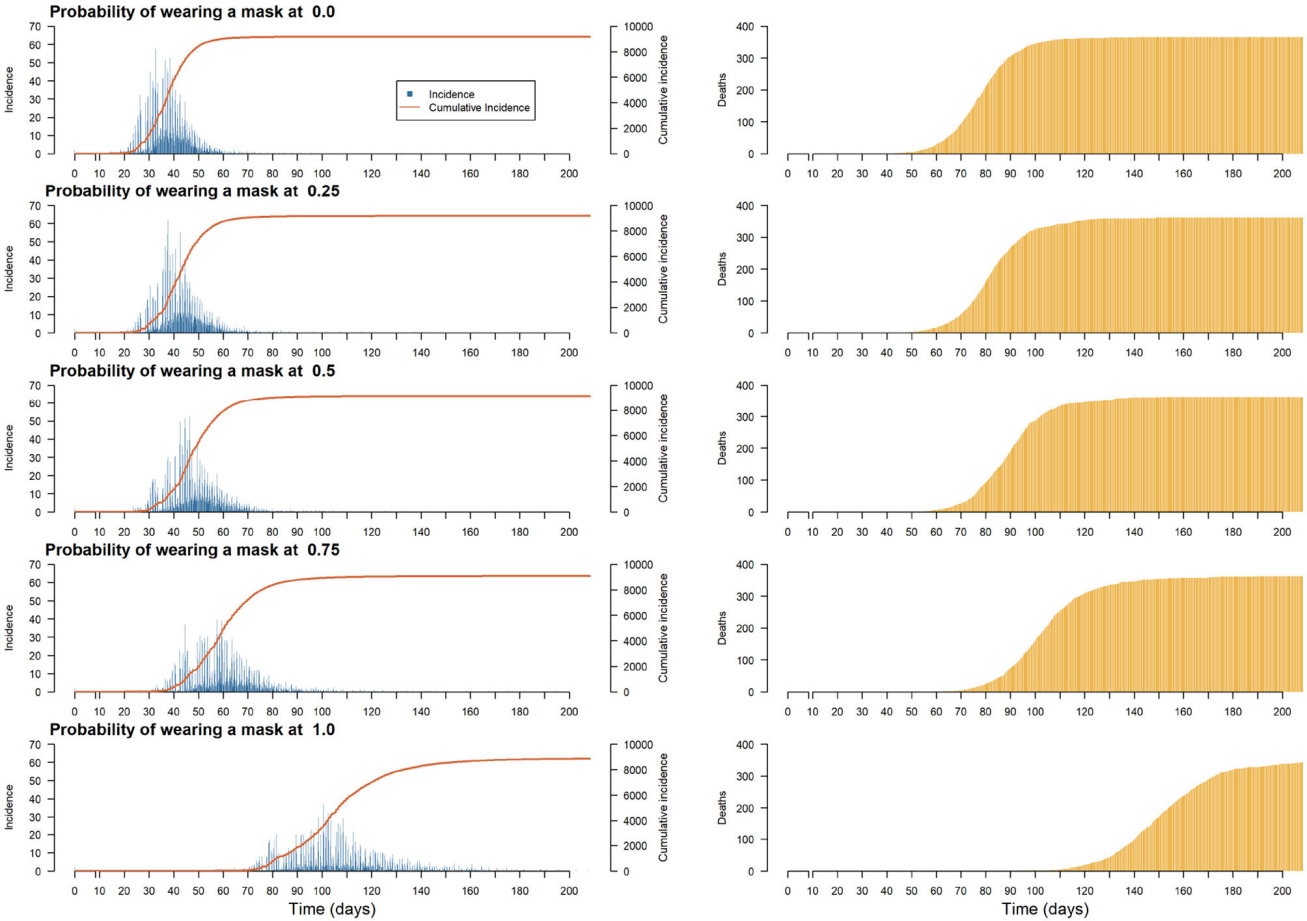
Plots of the median of the incidence and cumulative incidence and deaths on the population per step for different proportions of the population wearing a mask.

In Figure 7, even if wearing a mask does not help reduce the total number of infections or deaths (because it only influences disease transmission), it is found that the use of masks helps to flatten the incidence curve. Therefore, recommending the use of face masks would avoid overloading hospitals and intensive care units in our model as much as possible. The most important change in the dynamics of the incidence curve was achieved with a probability of wearing face masks of 0.75 (and above), which avoided the sudden increase in cases that was still noticeable with a probability of wearing face masks of 0.5. Since the policy applied was only to wear masks, no symptomatic individuals were admitted to hospital. Therefore, neither hospital overload nor the benefits of being admitted were simulated. The number of deaths did not change, but the reduction in the number of infected persons should avoid exceeding hospital capacity as much as possible.

#### 4.2.2. Impact of the Duration of the Lockdown on the Epidemic Peak

Faced with a pandemic without specific treatment or vaccine, public health services rarely have any choice but to choose policies to limit transmission and “flatten the curve” of incidence in the population. Depending on the country and region, there is a range of measures from soft social distancing, such as wearing masks in public spaces, avoiding congested areas or maintaining a distance of 1 m from other public transport users, to blanket travel restrictions, forced quarantine, total containment and technological monitoring (59). The positive effects of such actions on the number of hospitalizations, intensive care admissions, deaths or on the reproduction number have been demonstrated in different contexts, such as France (16, 60), Wuhan in China (61) or Italy (62). While complete lockdown appears as one of the best ways to mitigate the spread of an epidemic, it raises serious concerns related to economic (63) and socio-psychological (64) outcomes and also questions about the duration of its effectiveness (65) or the consequences of a partial or total lifting of restrictions (16).

The aim of this experiment is to evaluate the impact of the duration of a complete lockdown (i.e., when all the activities are forbidden) on the incidence, the number of recovered and dead Individuals. Simulations are launched with a simulation parameter encoding the duration of the lockdown taking values among 0 (no lockdown), 15, 30, 45, 60, and 90 days. More specifically, we observe how lockdown duration modifies the magnitude (e.g., lower or flatten) and time frame (e.g., happen fast or last long) of the epidemic peak. All the simulations are initialized with two new infected Individuals chosen randomly in the population. The complete lock-down policy is applied at the initial state of the simulation. The case study is also a simplified situation as no infected Individuals, external to the commune, can enter in the commune during the simulation.

**Figure 8:**
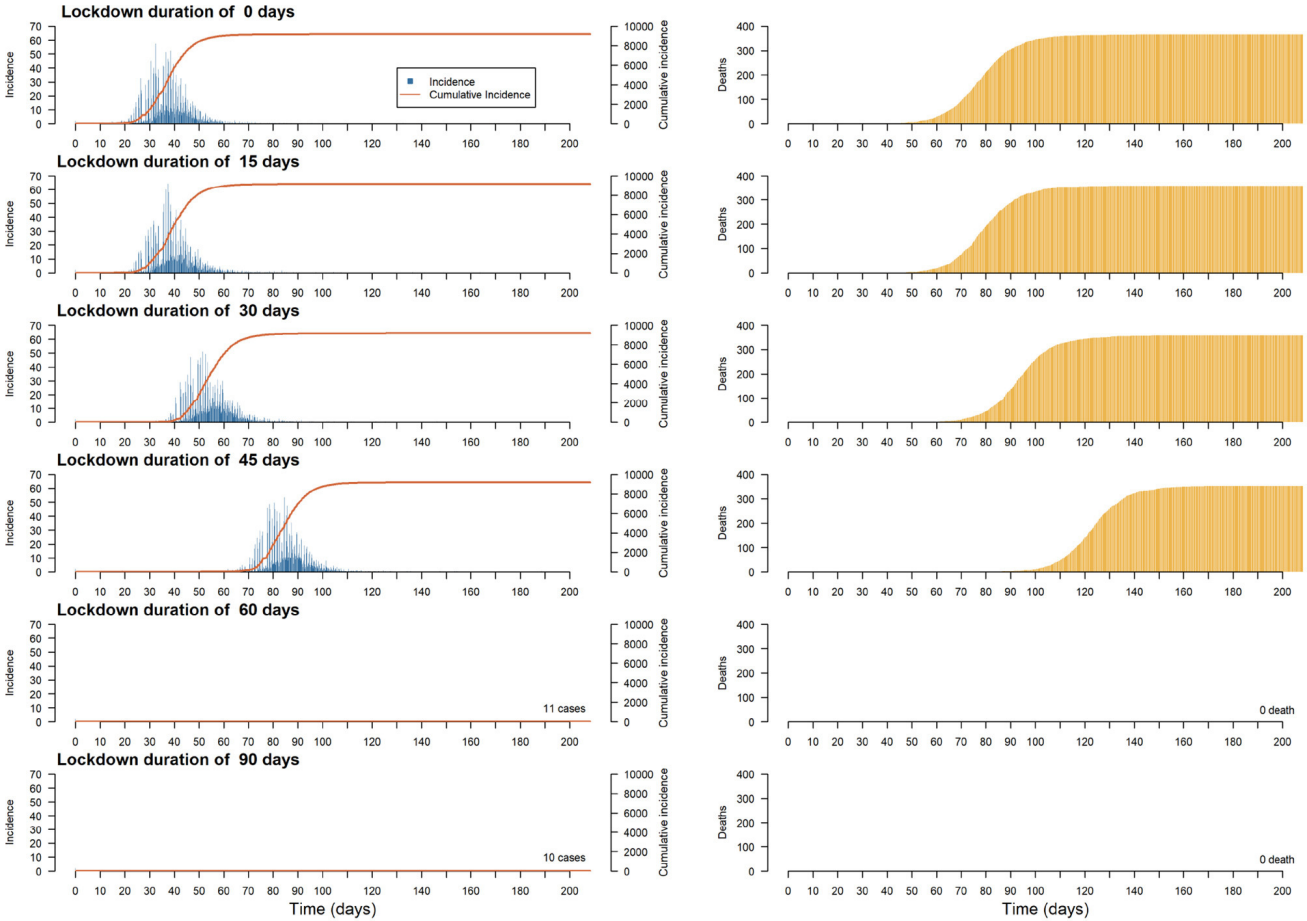
Plots of the median of the incidence, cumulative incidence and deaths on the population per step for different lockdown durations.

In Figure 8, we have plotted the incidence, cumulative incidence and deaths in the population, per step, for different lockup durations. First of all, we can see that it is not necessary to continue confinement after 60 days, as this is enough time to let the disease disappear. For shorter durations, preliminary results show that a peak in the number of infected individuals cannot be avoided, although confinement for between 15 and 45 days tends to delay the peak (by giving the health services more time to prepare) and flatten the curve (by avoiding overloading hospitals).

#### 4.2.3. Comparison of Realistic Policies

The objective of the last experiment is to compare the impact on the same case study of three realistic public health policies:

- *A combination of policies similar to that used in South Korea*: mass testing (in the model: more than 900 tests per day) with home quarantine for households with confirmed cases. South Korea is recognized as one of the countries with the most effective mitigation strategies implemented: according to the UNDP (3), it was one of the first countries to implement mass test programs (between 15 and 20,000 tests per day) with home quarantine guidelines for confirmed cases. The South Korean government's rapid and organized response has produced excellent results in freezing the early spread of the epidemic.
- *A combination of policies similar to the one used in France*: few tests (in the model: less than 200 per day) and, from 1% of confirmed cases, significant mobility restrictions applied to 90% of the population (to take into account people who cannot work at home and who are essential for everyday activities). According to the French government, there have been ~5,000 tests per day on average from the beginning of the epidemic, which is 4–5 times less than Korea or Germany. Regarding the lockdown, while it was one of the first countries to cancel major events, the closure of schools and non-essential economic activities occurred 14 days after the first deaths due to COVID-19, only preceding Great-Britain among the European countries (60).
- *A combination of policies similar to the one used in Malta*: no confinement for all the Individuals, but individuals belonging to risk groups (in the model: individuals over 50 years old) are required to stay at home.

**Figure 9:**
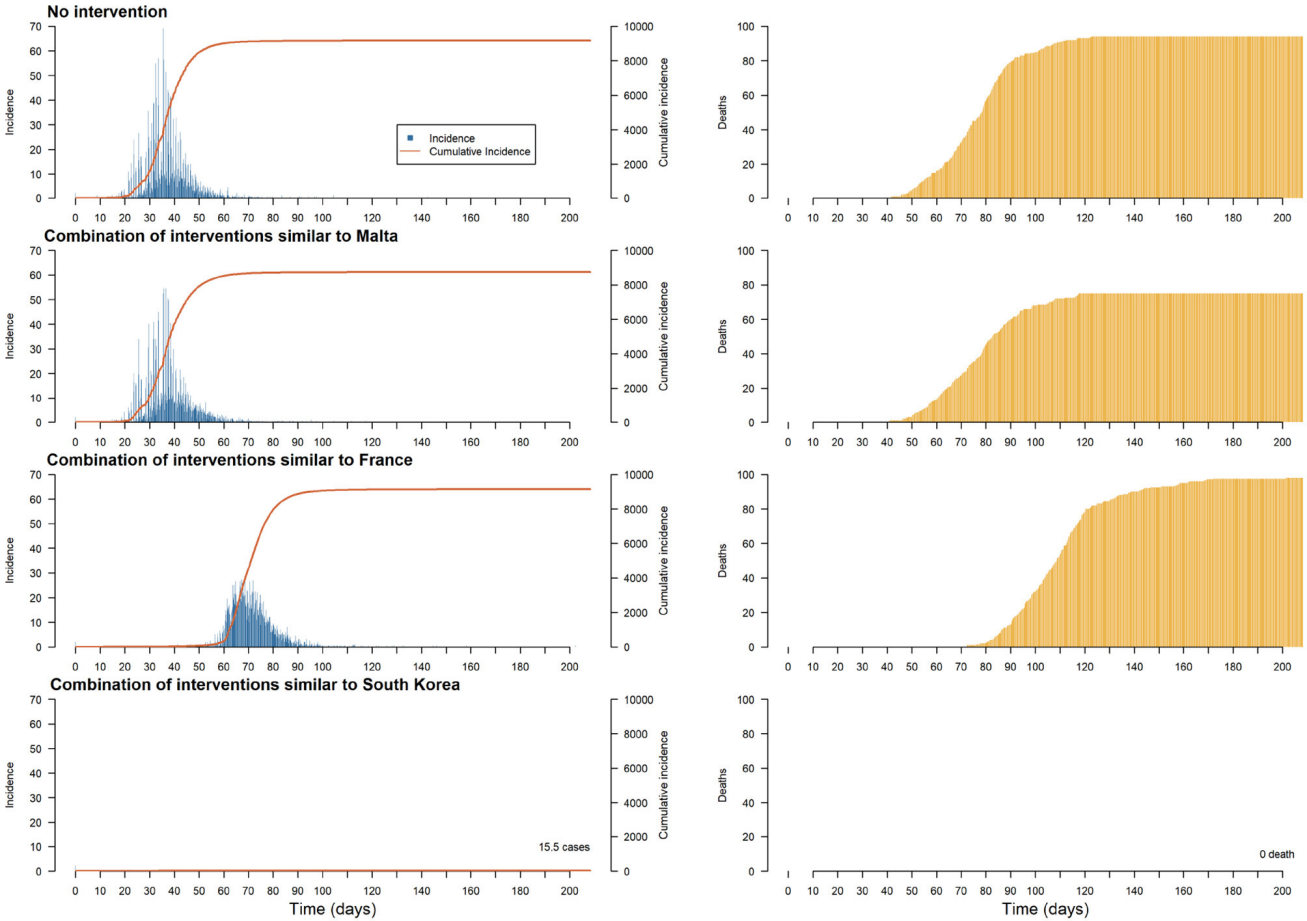
Plots of the median of the incidence, cumulative incidence and deaths on the population per step for different realistic interventions (each with a duration of 60 days).

In Figure 9, we depicted the policy consequences over the incidence (left column) and the number of casualties (right column). Only the policy involving a conjunction of mass testing and confirmed cases' household home confinement (similar to what South Korea implemented) have been able to contain the epidemic. However, the two other policies lead to specific mitigation outcomes: small sample testing in conjunction with heavy restriction on movement manages to delay and flatten the epidemic curve, while home containment directive toward at-risk people seems to lower the number of deaths.

All these experiences illustrate the characteristics and capabilities of COMOKIT. First of all, we have shown that, although the model is very stochastic, it is not very sensitive to this randomness, which allows us to launch explorations with a limited number (50) of replications. Second, we illustrated the ability of COMOKIT to compare different policies, for example by exploring the impact of the proportion of the population wearing a mask or the duration of a lock. Finally, we highlighted the expressive power of the model by implementing realistic policies close to those applied in three countries and by being able to compare their effectiveness at the scale and in the context of the Vietnamese commune used as a case study.

## 5. Conclusion and Perspectives

In less than 3 months after its emergence in China, the COVID-19 pandemic spread to the entire world. In the absence of prior experience with this new disease, public health authorities were forced to experiment, in a short period of time and in a largely uninformed manner, with various combinations of interventions at different scales.

As the pandemic continues its progression, data are being collected from a variety of sources, allowing authorities to make adjustments to ongoing and planned interventions, but also revealing an urgent need for tools and methodologies to quickly analyse, understand, compare, and predict the effectiveness of responses to COVID-19 in different communities and contexts. In this perspective, computer modeling, and especially agent-based approaches, allows detailed *in silico* exploration of these responses prior to their potential implementation. In this paper, we presented an agent-based modeling software built on the GAMA platform called Covid-19 Modeling Kit (COMOKIT), designed to be generic, scalable, and thus portable in a variety of social and geographical contexts.

COMOKIT is an integrated model, presented in detail in section 3, which combines a direct person-to-person transmission sub-model, an environmental transmission sub-model across the built environment, a policy design sub-model, and a person mobility and activity model based on a 1-h time step agenda. As shown in section 4, COMOKIT offers many guarantees in terms of reproducibility of results and sensitivity to input parameters. In addition, as we have demonstrated by implementing and comparing different policies and policy combinations, COMOKIT is modular and flexible enough to allow modelers to represent different strategies and study their impacts under several social, epidemiological or economic scenarios. It should be noted that although it comes with a predefined set of policies and activities for individual agents (e.g., buying, studying, working, etc.), adapted to most contexts, it can easily be extended to new agents, policies or activities by editing the models written in GAML.

Thanks to this inner flexibility and genericity, and to the increasing availability of open data, new case studies can be processed in COMOKIT within a few hours, allowing it to be used in a variety of contexts and by a majority of decision-makers. In fact, as shown in section 3, the model can work with only a minimal (usually open) initial dataset: the built environment and administrative boundaries of the study area can be extracted from OpenStreetMap, while a statistically consistent synthetic population can be generated by the Genstar Toolkit from IPUMS datasets. More accurate and sophisticated data can of course be mobilized to support the design of more complex models if required, and this can be done in a progressive and incremental fashion. This first version of COMOKIT (version v1.0, released in May 2020) has however some limitations that are already identified and that we think we can gradually remove with the help of other modelers:

- Scaling up: in computational terms, an agent-based approach will always be more expensive than an aggregate approach, not only in terms of execution time, but also in terms of the necessary replications (with respect to deterministic mathematical models). In its version 1.0, COMOKIT can reasonably (i.e., in less than 10 min on an average laptop with a graphical user interface enabled) simulate several months of pandemic fighting in cities with 10–20,000 inhabitants. Why take this standard? Quite simply because many users will test COMOKIT in this way and they should also be able to benefit from it. More serious experiments, varying more parameters and exploring different scenarios, will of course require scaling up. We are working on scaling up on two fronts: the first is to make it as easy as possible to use an HPC architecture from the simulator so that any user can access sufficient computing resources to run many replications or parallelize some of the operations of the simulations (66). This approach is the subject of a partnership with the EDF company, which has agreed to make its computing resources available (including the GAIA supercomputer); the second is to allow a more significant scaling-up of the model itself by implementing a hybrid approach (67–69) that is capable, dynamically, of aggregating individuals into groups of individuals according to different criteria (belonging to the same household, presence in the same space, sharing the same states, etc.) when this proves possible and relevant, in order to simulate much larger scales. As GAMA allows to couple computer models and mathematical models within the same simulation at different scales (70), this approach will not pose any technical problems, but it does raise quite interesting conceptual problems (71).
- The second limitation of the model is related to the assumptions made regarding the representation of group activities. So far, by design, no activities can be held outside a building and no group transportation is represented (for obvious reasons given the size of the initial case studies). This implies that agents cannot congregate outside buildings, nor can they congregate by chance; when they do congregate and have a chance to contaminate each other, it is because they are performing the same activity and/or are located in the same building. This strongly limits the representation of informal activities, such as markets or street restaurants, which are so common in Vietnam and other countries, outdoor public events (concerts, religious gatherings, etc.) or collective leisure activities (walks in pedestrian areas, parties, etc.), even though some of these activities (especially religious gatherings) are suspected to have contributed to the initial creation of clusters. Moving to larger scales will also, of course, require taking into account the transmission in public transport, from human to human during travel, but also through the environment, via the contamination of shared surfaces. These extensions are already planned for the next version of the model, but any new contribution is of course welcome!

The COVID-19 pandemic has resulted in countless casualties and contaminations, imposing massive public health campaigns, such as social isolation through widespread containment. The differences between countries and territories in terms of the occurrence of the virus and the number of victims are striking, as are the approaches of governments and their effectiveness in combating the pandemic. In such a context, it is important to recognize the increasing importance of data-based modeling approaches in the design of public health strategies. Platforms, such as COMOKIT can contribute to this effort, provided, as in this case, that they are open, transparent, easily explorable and testable, and above all built on sound theoretical and computational foundations.

## Data Availability Statement

The version of COMOKIT used in this paper is labeled V1.0. The source code of this version, together with the input dataset and all the experiments presented in this paper, are available in the repository of the project: https://github.com/COMOKIT/COMOKIT-Model. To be executed, COMOKIT requires GAMA, an open-source modeling and simulation platform, in its version 1.8.1. GAMA is available at: https://gama-platform.org. In order to make it as easy as possible for readers to run COMOKIT, an all-in-one release, including a Java virtual machine, the correct version of the GAMA platform and COMOKIT version 1.0 with all the datasets, is available here: https://github.com/COMOKIT/COMOKIT-Model/releases.

## Author Contributions

AD, BG, and PT were the principal designers of this document and were responsible for much of the writing. DP, NH, KC, and AB organized most of the experimental study to gather the study data and are responsible for the accuracy of data analysis and interpretation. PL performed the manuscript reviews. All authors have worked together, each in their own specialty, to design, and describe the research presented in the paper.

## Conflict of Interest

The authors declare that the research was conducted in the absence of any commercial or financial relationships that could be construed as a potential conflict of interest.

## Footnotes

^1^The complete description of the COMOKIT model, using the classical O.D.D. protocol (49), is available at the address: https://comokit.org/ressources/ODD-COMOKIT_v1.pdf.

^2^OSM stands for OpenStreetMap. The data have been accessed and retrieved from the website: https://www.openstreetmap.org/, using the provided Application programming interface (API).

^3^https://ipums.org/

^4^Consider the distribution tables of age and sex attributes in the real population and in the synthetic population. The TAE is “the sum of absolute differences between cells” in these two tables (51), and the normalized TAE is the TAE divided by twice the population size. This normalized TAE represents the rate of individuals with at least one incorrect value in a category.

^5^The method is detailed on the COMOKIT website: https://comokit.org/docs/parameterize#calibration-of-the-transmission-rate-value-beta.
